# Determinants of non-adherence to antiretroviral therapy among displaced people living with HIV and comparison with host populations: a cross-sectional study in Ituri and North Kivu

**DOI:** 10.11604/pamj.2025.52.8.40482

**Published:** 2025-09-08

**Authors:** Claude Ngona Mandro, Trésor Kashinde Mosomo, Serge Tonen Wolyec, Zacharie Tsongo Kibendelwa, Stanis Okitotsho Wembonyama

**Affiliations:** 1 Department of Public Health, Faculty of Medicine, University of Bunia, Bunia, Democratic Republic of Congo; 2 Department of Epidemiological Surveillance, Goma School of Public Health, University of Goma, Goma, Democratic Republic of Congo; 3 Department of Internal Medicine, Faculty of Medicine, University of Bunia, Bunia, Democratic Republic of Congo,; 4 Department of Internal Medicine, Faculty of Medicine, University of Kisangani, Kisangani, Democratic Republic of Congo,; 5 Department of Paediatrics, Faculty of Medicine, University of Lubumbashi, Lubumbashi, Democratic Republic of Congo

**Keywords:** Persons living with HIV, displaced, adherence, antiretroviral therapy

## Abstract

**Introduction:**

little information is available on the extent of HIV and Antiretroviral therapy (ART) adherence among displaced people living with HIV (PLHIV). The objective of this study was to compare the prevalence of ART non-adherence between displaced PLWHIV and the host population, and to identify determinants of non-adherence to ART among displaced PLHIV.

**Methods:**

a cross-sectional analytical study involving 444 adults living with HIV, both displaced persons and hosts, was conducted in Ituri and North Kivu from November 2022 to January 2023. Adherence was assessed based on patient reports and prescription refills. Logistic regression in SPSS 25 was performed to identify determinants of non-adherence to ART.

**Results:**

the prevalence of non-adherence was higher among displaced PLWHIV compared to those in the host population: 30% (24%-36%) vs 21% (18%-23%), p= 0.002. Non-adherence among displaced PLHIV was not different between the two provinces: 26% (18%-34%) in Ituri vs 34% (26%- 43%), p= 0.09 in North-Kivu. The main reasons for not taking ART among displaced PLWHIV were: forgetfulness, lack of food, occupation and travel. Multivariate analysis identified camp-to-hospital time greater than one hour (aOR: 2.32; CI 95%: 1.21 - 7.20), patient dissatisfaction (aOR: 6.20; 95% CI: 2.16 - 17.74), opportunistic infections (aOR: 5.32; CI 95%: 1.86 - 15.57), and stigma (aOR: 7.31; CI 95%: 2.82 - 18.84) as determinants of non-adherence.

**Conclusion:**

forced displacement favors non-adherence to ART. The specificities of displaced PLHIV require specific strategies to improve their retention on ART in order to reduce HIV-related morbidity and mortality.

## Introduction

The World Health Organization (WHO) recommends that anyone at risk of contracting the human immunodeficiency virus (HIV) should have access to a screening test, and treatment should be started as soon as a positive result is confirmed, regardless of their clinical condition or CD4 count, to prevent transmission of the disease to healthy people [1]. In 2022, in the world, 29.8 million people living with HIV (PLHIV) were receiving antiretroviral therapy, representing a global coverage of 76.4% [2]. To better control the HIV epidemic, UNAIDS has put in place the 95-95-95 strategy, which aims to ensure that 95% of people living with HIV know their serostatus, 95% have access to Anti-retroviral Therapy (ART) and 95% of those on treatment have an undetectable viral load 3]. Although some sub-Saharan African countries have already reached this target, ART coverage remains low, leaving almost 4.7 million PLHIV without treatment in 2023 [4,5]. By 2023 in the Democratic Republic of Congo (DRC), 84.69% of PLHIV knew their serostatus, 86% were receiving antiretroviral treatment and 77% had their viral load suppressed [6,7]. Adherence to antiretroviral treatment remains low in the DRC, as indicated by this study conducted in Kinshasa in 2023, which showed that only 55.6% of patients were adherent [8].

Poor adherence to treatment is one of the causes of treatment failure. Although the efficacy of ART relies on daily medication, the rigid schedule and lifetime duration of treatment make adherence difficult [9]. Little information is available on the extent of HIV among refugees, internal displaced people (IDP) and other crisis-affected people, and information on the need for ART for these populations is even less known [10]. Many studies on adherence to ART among PLHIV in the general population have identified non-disclosure of HIV status, stigma, not belonging to a support group, forgetfulness, lack of food, adverse effects, lack of money for transportation, stock-outs, and travel as factors in poor adherence to treatment [11-18]. Refugees, internally displaced persons and crisis-affected people are a specific category of populations for which few studies on ART adherence have been conducted. In a study of PLHIV in the Nakivale refugee camp in Uganda, the main barriers to adherence were difficulty in accessing the clinic, food insecurity, drug stock-outs, violence and unrest in the camp [19].

According to the experience of Doctors Without Borders on HIV treatment in the context of war, no difference was found in adherence compared to stable contexts [20], and a similar study conducted in Kenya reached the same conclusions [21]. In contrast, a systematic review of several studies examining ART adherence in conflict-affected and forcibly displaced populations showed adherence prevalence ranges of 87-99.5% [22]. Many studies have shown that adherence to antiretroviral treatment among people living with HIV in host populations remains a problem in HIV care, even though it is recognized that good adherence promotes viral load suppression. As vulnerable populations, refugees, internally displaced persons and people affected by crises are exposed to more problems related to antiretroviral treatment. However, in the DRC, little information is available on this subject [20]. We therefore conducted this study to estimate the level of adherence to antiretroviral treatment among displaced persons living with HIV, compare it with that of people living with HIV in host populations, and identify the determinants of adherence in order to develop targeted interventions aimed at improving adherence among people living with HIV who are displaced or refugees.

## Methods

A cross-sectional analytical study was conducted from November 2022 to January 2023 in Ituri and North Kivu provinces, in the eastern region of the DRC, among people living with HIV from host populations and internally displaced persons. Six sites for displaced persons were randomly selected from among the most densely populated sites established at least three months ago around or within the cities of Goma and Bunia.

**Study population:** the study population consisted of PLHIV who had been on treatment for at least three months and who were being cared for in the main facilities in the provinces of Ituri and North Kivu, as well as displaced persons in IDP sites in these two provinces and who were at least 18 years old.

**Inclusion criteria:** all PLHIV aged at least 18 years old, on ART for at least three months at the time of the study, and residing in areas of conflict and displacement (IDP sites and host communities in North Kivu and Ituri provinces) were included in this study.

**Exclusion criteria:** all PLHIV aged 18 years or older residing in conflict and displacement areas (IDP) sites and host communities in North Kivu and Ituri provinces) selected for the study but absent at two visits during the survey were excluded from the study.

**Sample size calculation and sampling techniques:** the sample size was calculated using the statcalc application of Epi info version 7.2 software according to the following formula.


$$N=\frac{DEFF*Np*p*\left(1-p\right)}{\frac{d^{2}}{Z_{1-\infty /2}^{2}}*\left(N_{p-1}\right)*p*\left(1-p\right)}$$

The following were used to calculate the sample size: DEFF: sampling plan correction factor; Np: size of target population (41,571); Z_α_: value of Z for the first species risk (for α = 5%, Z_α_= 1.96); p: estimated proportion of the population with the characteristic: 30.1% [23]; d: confidence limits: 5%. The calculated size was: 321. With a projected 20% non-response rate, the adjusted minimum sample size was 386. We surveyed 444 PLHIV, including 244 among displaced persons and 200 in host populations. In each category, the number of respondents was distributed equally between the two provinces. Respondents were selected at random using the random number generator in Open Epi 3.2 software from lists of patients identified in health facilities and displaced persons camps.

**Data collection:** data were collected from PLHIV who met the inclusion criteria and who had given informed consent. A structured and pre-tested questionnaire was administered face-to-face to the respondents and completed by reviewing the patients' charts. Adherence to ART was assessed on the basis of compliance with treatment during the seven days preceding the survey and compliance with prescription renewal appointments during the last three months preceding the survey. Patients were considered non-adherent if they reported missing at least one ART in the last seven days and/or missing at least one refill appointment in the three months prior to the survey [24]. The following independent variables were collected: sociodemographic (age, gender, employment status, marital status, religion, education level ( low level of education has been defined as less than a secondary school diploma, and a high level of education corresponded to at least a secondary school diploma), first camp for displaced, migratory status (e.g., displaced or host population); therapeutic and environmental characteristics (time from home to the site of care, patient satisfaction, duration of treatment, duration of diagnosis-beginning of treatment, the use of an alternative treatment, presence of opportunistic infections and stigma.

**Data processing and analysis:** the data were analyzed with Statistical Package for the Social Sciences (SPSS) software, version 25. Relative and absolute frequencies were calculated for the qualitative variables. The Kolmogorov-Smirnov test was performed to determine the normality of the data distributions for the quantitative variables. Normally distributed data were summarized as mean and standard deviation, and Student's t-test was used to compare means. Data that was not normally distributed was summarized as median and interquartile range. Pearson's chi-square test was performed to compare the proportions. A bivariate analysis was used to look for associations between the dependent variable and the independent variables one by one. Crude odds ratios with 95% confidence intervals and a p-value level of 0.05 were calculated for this purpose. All variables that showed a significant association in the bivariate analysis (p < 0.05) were included in the binary multiple logistic regression model. The Hosmer-Lemeshow test was performed to assess the quality of the model. The model was considered good when the p-value was greater than 0.05. At the end of the multivariate analysis, variables with a p-value < 0.05 were retained as determinants of non-adherence to ART. For each independent variable retained, we calculated the adjusted odds' ratio (aOR) with its confidence interval at 95% and the p-value at the significance level (< 0.05)

**Bias:** a potential memory bias could have come into play because some questions concerned events dating back a few days, such as the reasons for not taking ART, or information bias could have been due to incorrect classification of respondents as compliant or non-compliant. Memory bias was controlled as much as possible by asking questions in such a way that respondents could recall past events as accurately as possible, and information bias was controlled by using clear definitions to ensure that respondents were correctly classified.

**Ethical considerations:** approval from the University of Goma committee was obtained under number UNIGOM/CEM/006/2022. All respondents duly signed an informed consent form, translated into the language they best understood. Participation in the interview was voluntary and confidentiality of information collected from respondents was guaranteed.

## Results

**Prevalence of non-adherence to ART:** the prevalence of non-adherence was higher among displaced PLHIV than among the host population: 30% (24%-36%) vs 21% (18%-23%), p= 0.002. The prevalence of non-adherence among displaced PLHIV was not different between the two provinces: 26% (18%-34%) in Ituri vs 34% (26%- 43%), p= 0.09 in North Kivu.

**Sociodemographic characteristics of displaced PLHIV in Ituri and North Kivu:** a total of 244 displaced PLHIV took part in the survey. The majority of respondents (48%) were aged between 25 - 35 and 57% were women. More than half of the respondents (59%) were unemployed, and nearly three-quarters (72%) were married. In addition, PLHIV with a high level of education (67%), and those who were in their first IDP camp were the most represented (Table 1).

**Table 1:** sociodemographic characteristics of displaced persons living with HIV in Ituri and North Kivu, 2023

Variables	Frequency		Proportions
**Age groups (years)**			
18 - 24	22		9
25-35	117		48
36 - 45	96		39
≥ 46	9		4
**Sex**			
Female	138		57
Male	106		43
**Professional situation**			
Unemployed	144		59
Employed	100		41
**Marital status**			
Single	69		28
Married	175		72
**Religion**			
None	7		3
Church of revival	84		34
Catholic	67		27
Protestant	82		34
Other	4		2
**Educational level**			
Low level	81		33
High level	163		67
**First camp for displaced**			
No	98		40
Yes	147		60

**PLHIV**: Persons Living with Human Immunodeficiency Virus

**Therapeutic and environmental characteristics of displaced PLHIV:** the majority of PLHIV (16%) took at least an hour to reach the health care facility where they obtained ART, 90% were satisfied with the health care services they received, 9% had opportunistic infections, 4% said they had already used an alternative treatment to ART and 14 % had already been the victim of stigmatization by those around them (Table 2). The mean duration of treatment did not differ between displaced PLHIV in Ituri (24.3 +/-22.4 months) and those in North Kivu (20.8 +/- 19.2 months), (p=0.129). The average time since diagnosis did not differ between displaced PLHIV in Ituri (26.5 +/-24.5 months) and those in North Kivu (22.6 +/-19.2 months), (p=0.157).

**Table 2:** therapeutic and environmental characteristics of displaced PLHIV in Ituri and Nord-Kivu, 2023

Variables	Frequency	Proportions
**Home-hospital time (Hours)**		
≥ 1	39	16
< 1	205	84
**Patient satisfaction**		
No	21	10
Yes	200	90
**Opportunistic infections**		
Yes	21	9
No	223	91
**Alternative Treatment to ART**		
Yes	9	4
No	235	96
**Stigma**		
Yes	35	14
No	209	86

**PLHIV:** Persons Living with Human Immunodeficiency Virus; ART: antiretroviral therapy

**Figure 1:**
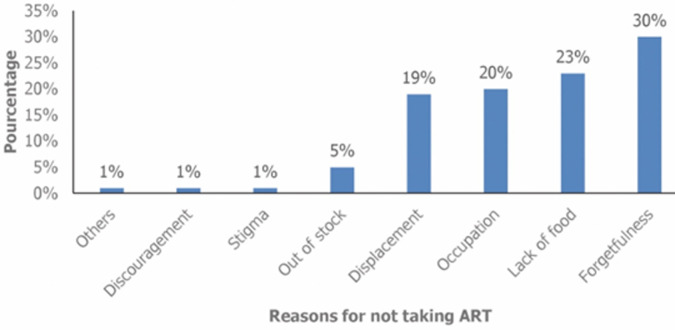
reasons for not taking antiretroviral treatment among persons living with HIV displaced in North Kivu and Ituri, 2023

**Determinants of non-adherence to ART:** after adjusting of variables with statistically significant associations in bivariate analysis, travel time between place of care and hospital of one hour or more (aOR 2.32; 95% CI: 1.21-7.20, p= 0.017), patient dissatisfaction with the organization of care (aOR 6.20; 95% CI: 2.16 - 17. 74, p= 0. 001), the presence of opportunistic infections (aOR 5.32; 95% CI: 1.86 - 15.57, p= 0.002) and stigma (aOR 7.31; 95% CI: 2.82 - 18.84, p < 0.001) were factors independently associated with non-adherence to antiretroviral treatment among PLHIV displaced to IDP sites in Ituri and North Kivu provinces (Table 3). Forgetfulness (30%), lack of food (23%), occupation (20%), and displacement (19%) were the most frequently cited reasons for not taking ART by PLHIV (Figure 1).

**Table 3:** determinants of non-adherence to antiretroviral therapy among displaced PLHIV in Ituri and Nord-Kivu, 2023

Variables	Non-adherence n (%)	Adherence n (%)	c OR	CI 95%		P-value	AOR	CI 95%		P-value
**Sex**										
Female	43(31)	95(69)	1.14	0.65	2.01	0.633				
Male	30 (28)	76(72)	1							
**Professional situation**										
Unemployed	51(35)	93(65)	1.93	1.08	3.52	0.024	0.83	0.38	1.80	0.637
Employed	22(22)	78(78)	1				1			
**Marital status**										
Single	29 (42)	40 (58)	2.15	1.19	3.88	0.011	0.86	0.36	2.03	0.738
Married	44 (25)	131 (75)	1				1			
**Educational level**										
Low level	36(44)	45(56)	2.71	1.53	4.83	<0.001	1.55	0.70	3.41	0.279
High level	37(30)	126(70)	1				1			
**First camp for displaced**										
No	18(17)	80(83)	0.36	0.19	0.66	<0.001	1.63	0.75	3.53	0.217
Yes	56(38)	90(62)	1				1			
Home-hospital time (Hour)										
≥ 1	23(59)	16(41)	4.42	2.16	9.18	<0.001	2.32	1.21	7.20	0.017
< 1	50(24)	155(76)	1				1			
**Patient satisfaction**										
No	15(71)	6(29)	8.50	3.17	25.12	<0.001	6.20	2.16	17.74	0.001
Yes	45(23)	155(77)	1				1			
**Opportunistic infections**										
Yes	13(62)	8(38)	4.38	1.73	11.64	0.001	5.32	1.86	15.57	0.002
No	60(27)	163(73)	1	1			1			
**Alternative Treatment**										
Yes	8(89)	1(11)	20.66	3.22	470.30	<0.001	7.32	0.88	60.21	0.961
No	65(28)	170(72)	1				1			
Stigma										
Yes	25(71)	10(29)	8.29	3.77	19.25	<0.001	7.31	2.82	18.84	<0.001
No	48(23)	161(77)	1				1			

PLHIV: persons living with human immunodeficiency virus; CI: confidence interval; OR: Crude: OR; AOR: Adjusted: OR

## Discussion

The objectives of this study were to compare the level of ART adherence between displaced and host PLHIV and to identify the determinants of non-adherence to ART among displaced PLHIV.

**Prevalence of non-adherence to ART:** in our study, displaced people living with HIV were less adherent. Studies conducted by Mendelsohn in Malaysia and Kenya, Garang in Uganda, and Spiegel's systematic review in sub-Saharan Africa yielded results contrary to ours, with no difference observed between these two categories [25-27]. This difference with us could be explained by the existence of support mechanisms for displaced PLHIV in their context, which is not the case in the DRC.

**Sociodemographic characteristics of displaced PLHIV in Ituri and North Kivu:** unemployment showed an association with non-adherence to ART. Similar results were obtained in Nigeria by Chime OH and Fikadu Tadesse Nigusso in Ethiopia [16,28]. Regarding marital status, being single was associated with non-adherence as also found by Vandana Hiregoudar in India [29]. Living with a partner could promote better adherence as the spouse can be a source of support for taking the medication and reminding them if they forget. As found by Bukenya D. in Uganda, we also found that low education level was associated with non-adherence [30]. Certainly, a high level of education facilitates better knowledge of the disease and the importance of adherence to treatment.

**Environmental characteristics of displaced PLHIV:** a time equal to or greater than one hour to reach the care site doubled the risk of non-adherence. Respondents living an hour or more from their drug supply site were less adherent. In fact, the long distance is a demotivating factor for some to go to their appointments. Mendelsohn JB in his study conducted simultaneously in Kenya and Malaysia came to a similar conclusion [25]. Patient dissatisfaction increases the risk of non-adherence. It turns out that when patients are satisfied with the services provided by providers, they are more confident about going to the facility for treatment renewal [26,31-34]. Stigma has been identified as a risk factor for non-adherence. Indeed, the fear or the fact of being shunned by relatives constitutes a real obstacle to adherence. Similar results have been found by other authors [9,11,35-42].

**Therapeutic and clinical characteristics of displaced PLHIV:** the use of alternative treatment was associated with non-adherence. This result is corroborated by Izizag *et al*. in the study conducted in Kinshasa [17]. In fact, the use of an alternative treatment proves that the patient is not totally convinced of the therapeutic virtues of ART, which results in the temporary or definitive abandonment of this treatment in favor of other therapies. The presence of opportunistic infections was identified in our study as a factor in poor adherence to treatment. Indeed, patients with opportunistic infections were more likely to be non-adherent with treatment. Nigusso and Chanie in Ethiopia found similar results. The treatment of opportunistic infections increases the number of drugs to be taken, which discourages patients and leads to poor compliance [28,36].

**Reasons for not taking ART:** forgetfulness, lack of food, occupation and displacement of PLHIV were the reasons for non-adherence mentioned by the displaced PLHIV. The precariousness of the living conditions of displaced persons is probably at the root of several reasons for non-adherence to treatment as found in our study and by other authors. Indeed, the lack of food and the displacement of the living environment do not favor the taking of treatment [43].

**Strength of the study:** this study is one of the few to have been conducted among displaced persons in the DRC. It highlighted the factors contributing to non-adherence to antiretroviral treatment among displaced persons and will enable appropriate measures to be taken to improve adherence among this group of people living with HIV. The representativeness of the selected sites allows the results to be generalized to all sites in these two provinces.

**Study weakness:** given that adherence was assessed based on the patient's self-assessment, an information bias could be introduced due to the subjectivity of the respondent's response.

**Limitations:** the cross-sectional nature of the study does not allow for the establishment of a temporal sequence between exposure and outcome. The absence of biological measures is a limitation because it does not allow for an objective assessment of non-adherence.

## Conclusion

Based on the results obtained, we can conclude that displaced persons living with HIV are less compliant than those in host populations. The long time to reach the care facility, the lack of satisfaction of patients with the service provided by providers, the presence of opportunistic infections, and stigma of the patient were identified as determinants of non-adherence to ART among PLHIV displaced in the provinces of Ituri and North Kivu. Improving the quality of life and health status of PLHIV requires good adherence to ART, which is the key to viral suppression. As displaced persons are a particular category of PLHIV, we recommend setting up care sites around the camps in order to reduce the distance PLHIV have to travel to obtain ART. A better patient-caregiver relationship will improve patient satisfaction, resulting in better treatment adherence, and we encourage HIV awareness among PLHIV and the community as well as teaching PLHIV to accept their condition and come out of clandestinity.

### What is known about this topic

- Lack of money encourages no-shows at prescription renewal appointments;
- Food insecurity is the main cause of non-adherence to treatment among persons living with HIV in mobile situations;
- Difficulty in accessing health care centers favors non-adherence to treatment among persons living with HIV in mobile situations.

### What this study adds

- The prevalence of non-adherence to antiretroviral therapy is higher among displaced persons: 30% (24%-36%) vs 21% (18%-23%);
- Displaced status is a factor favoring non-adherence to antiretroviral therapy;
- The long distance to travel to reach health care facilities favors non-adherence to antiretroviral therapy treatment.
